# Synthesis of a Curing Agent Derived from Limonene and the Study of Its Performance to Polymerize a Biobased Epoxy Resin Using the Epoxy/Thiol-Ene Photopolymerization Technique

**DOI:** 10.3390/polym14112192

**Published:** 2022-05-28

**Authors:** Ricardo Acosta Ortiz, Rebeca Sadai Sánchez Huerta, Antonio Serguei Ledezma Pérez, Aida E. García Valdez

**Affiliations:** Centro de Investigación en Química Aplicada, Blvd Enrique Reyna No. 140, Saltillo ZC 25294, Coahuila, Mexico; rebeca.sanchez.m20@ciqa.edu.mx (R.S.S.H.); antonio.ledezma@ciqa.edu.mx (A.S.L.P.); aida.garcia@ciqa.edu.mx (A.E.G.V.)

**Keywords:** limonene, curing agent, epoxy/thiol-ene, photopolymerization, kinetics

## Abstract

This study describes the synthesis of a curing agent derived from limonene as well as its application to prepare biobased thermoset polymers via the epoxy/thiol-ene photopolymerization (ETE) method. A biobased commercial epoxy resin was used to synthesize a crosslinked polymeric matrix of polyether-polythioether type. The preparation of the curing agent required two steps. First, a diamine intermediate was prepared by means of a thiol-ene coupling reaction between limonene and cysteamine hydrochloride. Second, the primary amino groups of the intermediate compound were alkylated using allyl bromide. The obtained ditertiary amine-functionalized limonene compound was purified and characterized by FTIR and NMR spectroscopies along with GC-MS. The curing agent was formulated with a tetrafunctional thiol in stoichiometric ratio, and a photoinitiator at 1 mol % concentration, as the components of a thiol-ene system (TES). Two formulations were prepared in which molar concentrations of 30 and 40 mol % of the TES were added to the epoxy resin. The kinetics of the ETE photopolymerizations were determined by means of Real-Time FTIR spectroscopy, which demonstrated high reactivity by observing photopolymerization rates in the range of 1.50–2.25 s^−1^ for the epoxy, double bonds and thiol groups. The obtained polymers were analyzed by thermal and thermo-mechanical techniques finding glass transition temperatures (Tg) of 60 °C and 52 °C for the polymers derived from the formulations with 30 mol % and 40 mol % of TES, respectively. Potential applications for these materials can be foreseen in the area of coatings.

## 1. Introduction

Currently, one of the main trends in research and development is oriented toward the development of new polymeric materials by utilizing raw materials derived from natural products [1,2,3]. Therefore, new biobased polymers are being developed with the aim to compete in cost and performance with the materials obtained from petrochemicals. Within the area of thermoset polymers, one of the priorities is the investigation of novel biobased epoxy monomers as well as new curing agents [4,5,6]. Some biobased thermosetting polyethers depict similar or superior properties to the polymers derived from the commercial epoxy resin diglycidyl ether of bisphenol A, which has the highest cost-benefit ratio in the global market [7,8,9,10,11,12,13]. The synthesis of novel biomass-derived curing agents for epoxy resins has also been studied and documented [14,15,16,17,18].

Limonene is a low-cost and readily available terpene with a variety of applications in different areas such as food and beverages [19,20], pharmaceutical [21], bio-solvent [22], cosmetics and personal care [23,24]. Limonene has also been used as a building block to prepare various types of biobased polymers [25,26,27,28]. This terpene is obtained from distillation or by solvent extraction from the peels of citrus fruits. The global production of citrus was found to be 98 million metric tons in 2020/2021 [29] with a fraction destined for industrial use [30]. The expected annual growth rate of the production of limonene is forecasted to be 4.5% in the 2021–2028 period [31].

Limonene is found in nature as (+)-*R*- and (−)-*S*-limonene enantiomers due to the presence of a chiral center in its structure [32,33]. The pair of double bonds in limonene allows the introduction of different functional groups. For instance, several methods have been used to obtain dihydroxylated and diaminated limonene derivatives [34], halogenated limonene [35,36] and limonene functionalized with heterocycle moieties [37]. Fuscalfo et al. described the synthesis of hydroxy-aromatic amines derived from limonene [38]. The epoxidation of limonene and further carbonylation, produced the polymerized carbonate to obtain the completely biobased poly (limonene carbonate) [39]. In the field of thermoset polymers derived from epoxy resins, limonene has been subjected to epoxidation reactions to obtain epoxy monomers that can be cured thermally or photochemically [40,41,42]. Additionally, multifunctional epoxy resins have been prepared by reacting limonene oxide with tri or tetrathiols via a thiol-ene coupling reaction [43]. Limonene has also been utilized as a starting material to produce a diamine-functionalized curing agent which is used to cure resorcinol epoxy resins [44].

Our research group has developed a cost-effective and ecological process to cure epoxy resins, known as the epoxy/thiol-ene photopolymerization (ETE) procedure. In this method, the anionic ring-opening polymerization (AROP) of the epoxy resin is combined with a thiol-ene photopolymerization [45]. The thiol-ene system (TES) comprises a multifunctional thiol, a curing agent of the ditertiary amine type and a photoinitiator. This TES can be added to the epoxy resin in concentrations ranging from 20 to 40 mol %. The curing agent used in this technique simultaneously induces both types of polymerization as a function of its allyl-functionalized tertiary amine groups. It was found that the epoxy groups as well as the thiol and double bonds of the TES, achieved quantitative conversions in less than 10 min by employing this procedure [46], whereas the conventional thermal curing of epoxy resin, requires longer times (6–12 h) and higher temperatures (100–180 °C) [47,48]. The photopolymerization method, therefore, demonstrates considerable energy efficiency, resulting in a more economical and ecological process compared to thermal curing. A covalently bonded crosslinked polyether-polythioether co-network is obtained, which displays enhanced toughness due to the presence of the flexible polythioethers [49]. This represents a technological advantage over the polyethers derived from the conventional thermal curing of epoxy resins that are highly brittle materials due to the increased level of crosslinking.

The high reactivity and versatility of the ETE photocurable system have allowed us to develop smart materials. For instance, the soft phase (polythioethers) and the hard phase (polyethers) in the co-network, lead to the production of shape-memory materials. These materials are able to recover their permanent shape in less than two seconds, when their temporary shape is subjected to a temperature exceeding their Tg [50]. In another study, the partial oxidation of the tetrafunctional thiol used in the ETE formulation was investigated to obtain an oligomer with thiol and disulfide groups in its structure. This species was introduced in the formulation instead of the multifunctional thiol to obtain the disulfide-functionalized polyether-polythioether crosslinked polymer which induces self-healing properties [51].

In another application of this technique, a foaming agent such as the benzenesulfonyl hydrazide was added to the photocurable formulation to obtain rigid epoxy foams with a controlled size of pores and improved toughness [52]. In addition, the development of fiber-composite materials with either glass-fiber [53] or carbon-fiber [54] has also been reported. Graphene oxide nanocomposites were also prepared with the help of this photopolymerization technique [55]. 

In an effort to obtain “green” materials using this ETE photopolymerization we synthesized biobased epoxy resins derived from nopol, a terpenoid derived from β-pinene, and studied their reactivity as well as the thermal and mechanical properties of the derived polymers. These monomers displayed high reactivity when photopolymerized, based on the highly strained cycloaliphatic epoxy groups [56]. In another investigation, we also prepared a novel curing agent bioinspired by cystamine, that could be used in the ETE photopolymerization. The aim of developing this curing agent was to introduce disulfide groups in the crosslinked co-network with the goal to impart self-healing properties to the materials [57]. 

Thus, by continuing with this research, we aimed to obtain biobased crosslinked polymers by using a rapid and efficient method of photopolymerization in this study. A novel curing agent derived from limonene was prepared to be used as a component of the TES, in conjunction with a commercial biobased epoxy resin. The kinetics of photopolymerization were determined using a time-resolved FTIR spectroscopy technique. The photocurable formulations were cured in a UV chamber and the obtained crosslinked co-networks were characterized by thermal and thermo-mechanical analysis. 

## 2. Experimental 

### 2.1. Materials 

(R)-(+) Limonene technical 90% (sum of enantiomers), cysteamine hydrochloride (98%), ethanol anhydrous (99.5%), 2,2-dimethoxy-2-phenyl acetophenone (99%) [DMPA], (3-mercaptopropyl) trimethoxysilane (95%) [MPTS], phenylbis-(2,4,6-trimethylbenzoyl)-phosphine oxide (99%) [BAPO], allyl bromide (99%), tetrabutyl ammonium bromide (99%) [TBAB], potassium iodide (99%), pentaerythritol tetrakis (3-mercaptopropionate) (95%) [PTKMP], hydrochloric acid (37%) and sodium hydroxide (98%) were purchased from Sigma-Aldrich Toluca, México, and were used as received. The biobased epoxy resin SR GreenPoxy 28 was purchased from Sicomin Epoxy Systems, Princeton, NJ, USA. This epoxy resin belongs to the glycidyl aromatic type with an epoxy equivalent of 195–204 g/eq.

### 2.2. Synthesis of 2-((2-(3-((2-Aminoethyl) thio)-4Methylcyclohexyl) propyl) thio) ethanamine (LC)

The method reported by Stemellen et.al [58] to introduce amino groups in the grapeseed oil was modified and utilized in this study to prepare the aminated limonene intermediate (LC). A 250 mL Erlenmeyer flask with a glass stopper was charged with 150 mL of absolute ethanol followed by 10 g (0.65 moles) of limonene, 44.78 g cysteamine hydrochloride (0.39 moles) and 1.38 g (3.2 × 10^−3^ moles) of BAPO. Subsequently, the mixture was stirred until the solid was completely dissolved. It was then irradiated with a Bluewave 200 Dymax UV-Vis lamp (Torrington, CT, USA) using a UV light intensity of 150 mW/cm^2^ for 8 h. After this time, the solvent was roto-evaporated, and the residue was cleansed with hexane to remove any unreacted limonene. This residue was subsequently re-dissolved in 50 mL of chloroform. This organic solution was then extracted with an aqueous solution of sodium hydroxide 10% p/v (4 × 30 mL), in order to remove the excess cysteamine hydrochloride. The desired product was obtained in quantitative yield as a clear dense liquid. The chromatogram obtained by GC-MS revealed the product to be a mixture of two enantiomers as shown in Figure S1. 

FTIR liquid film on KBr disc (cm^−1^): 3352 (-NH_2_), 2908 (–C-H), 1585 (C-N), 1449 (CH_2_). ^1^H NMR (500 MHz, CDCl_3_, δ ppm): 2.60 (m, 4H, -N-CH_2_-CH_2_-S-); 2.34 (m, 4H, -N-CH_2_-CH_2_-S-), 2.08 (m, 3H, -CH-S-CH_2_, -CH_2_-S-CH_2_-); 1.80–0.98 (m, 8H, cycloaliphatic H); 1.21 (s, 4H, -NH_2_), 0.85 (d, 1H, -CH-CH-), 0.77 (d, 3H, -CH_3_), 0.71 (d, 3H, -CH_3_).

GC-MS analysis: RT = 15.63 min, 15.69 min (two enantiomers), *m*/*z* = 273 (-NH_2_)

### 2.3. Synthesis of N-allyl-N-(2-((2-(3-((2-(Diallylamino)ethyl)thio)-4-Methyl cyclohexyl) propyl) thio) ethyl)prop-2-en-1-amine (LCA)

A 250 mL three-necked round bottom flask fitted with a condenser, thermometer and magnetic stirring was charged with 150 mL of toluene and 8.0 g (0.27 moles) of LC. The allyl bromide 23.31 g (0.19 moles) was added dropwise to the reaction mixture, followed by 7.71 g (0.19 moles) of powdered sodium hydroxide. Thereafter, 0.62 g (1.92 × 10^−3^ moles) of the phase transfer catalyst TBAB was added to the reaction mixture along with 0.32 g (1.92 × 10^−3^ moles) of potassium iodide as a catalyst. The mixture was heated at reflux temperature (105 °C) for 24 h. The resultant mixture was washed with distilled water (3 × 30 mL) and then dried using 2.0 g of anhydrous sodium carbonate. The residue was purified by flash column chromatography using a mixture of 95:5 ratio hexane to ethyl acetate as an eluent. The product was obtained as a clear liquid at a 90% yield. 

FTIR liquid film on KBr disc (cm^−1^): 3078 –C=C-, 2925 (-C-H), 2875 (C-H), 1638 (-C=C-H), 1585 (C-N) 1455 (-CH_2_-), 920 (-C=C).

^1^H NMR (500 MHz, CDCl_3_, δ ppm): 5.82 (m, 4H, -CH_2_-CH=CH_2_); 5.13 (m, 8H, -CH_2_-CH=CH_2_); 3.09 (m, 8H, -CH_2_-CH=CH_2_); 2.63 (m, 4H, -N-CH_2_-CH_2_-S-); 2.53(m, 4H, -N-CH_2_-CH_2_-S-, 2.37 (m, 1H, -CH-S-CH_2_); 2.25 (m, 2H, -CH_2_-S-CH_2_); 1–98–1.02 (m, 9H, cycloaliphatic H); 1.02 (d, 1H, -CH-CH-); 0.96 (d, 3H, -CH_3_), 0.90 (d, 3H, -CH_3_).

GC-MS analysis: RT = 21.75 min, 21.87 min (two enantiomers), *m*/*z* = 450 (mol ion peak), 409 (450-(-CH_2_-CH=CH_2_)

### 2.4. Determination of the Kinetics of the ETE Photopolymerization Using the Real-Time Fourier Transform Infrared Spectroscopy (RT-FTIR)

Table 1 displays the amounts in grams and in moles of the components of the photocurable formulations including the biobased epoxy resin SR GreenPoxy 28, the curing agent LCA, and the multifunctional thiol PTKMP and the radical photoinitiator DMPA. The PTKMP was added until the end to avoid premature crosslinking of the formulation. A drop of this formulation was placed between two corona-treated PP films of 2 cm × 2 cm. 

The sandwich was placed in a heated solid transmission cell and the temperature was increased to 85 °C. The intensity of the UV light was 40 mW/cm^2^. A Nicolet 6700 Thermofisher FTIR spectrometer (Franklin, MA, USA) was used which has the ability to run successive scans in a predetermined time. The tip of the light guide that emits the radiation produced by the Blue Wave 200 Dymax UV-Vis lamp (Torrington, CT, USA), was placed at an angle of 45 ° with respect to the horizontal infrared beam of the spectrometer, focusing the UV light beam at the center of the sandwich encasing the sample. The samples were irradiated with unfiltered UV light in the range of 300–450 nm. The scanning of the sample began as soon as the UV lamp was turned on. The equipment was set to run 1 scan/s for 600 s. Each sample was run a minimum of three times and the average is reported. The decrease in the absorbance of the bands at 2552 cm^−1^, 1646 cm^−1^ and 759 cm^−1^, characteristics of the thiol, double bonds and epoxy groups, respectively, were monitored during the analysis to obtain the conversion versus time curves of each functional group with the help of the following equation:

(1) $$\mathrm{Conversion}\left(x\right)=\left[\frac{A_{0}-A\left(x\right)}{A_{0}}\right]*100$$

where A_0_ is the initial absorbance and A_x_ is the absorbance at a given time

The photopolymerization rate was determined as the slope of the linear portion of the conversion-time curves. 

### 2.5. Bulk Photopolymerization of the ETE Formulation 

The photocurable formulation with the biobased epoxy resin and the TES was prepared to obtain the crosslinked co-network. In these experiments, the amounts in grams of each component were ten times more than those shown in Table 1 for the kinetics of photopolymerization.

A stainless-steel mold with a cavity area of 40 mm length, 10 mm width and 2 mm thickness, was used to produce the test specimens. The mold containing the samples was introduced in a vacuum chamber for 10 min to eliminate the air bubbles, followed by the introduction to a UV chamber provided with a Fusion 300 W microwave-started UV lamp. Each specimen was irradiated for 10 min on each side. As a result of the heat released by the UV lamp, the temperature inside the chamber was noted to be 85 °C. The UV light intensity reaching the sample was measured to be 40 mW/cm^2^. Following the irradiation time, the samples were allowed to cool down before being demolded and analyzed. 

### 2.6. Differential Scanning Calorimetry (DSC)

The thermal properties of the prepared composites were analyzed by the DSC technique using a TA Instruments Discovery 2500 differential analysis calorimeter (New Castle, DE, USA). Samples of 8–10 mg were weighed accurately and placed in the sample compartment. The equipment was set to heat the samples from 0 °C to 150 °C at a heating rate of 5 °C/min in a nitrogen atmosphere. The Tg was determined from the second heating curve.

### 2.7. Dynamic Mechanical Analysis (DMA) 

The obtained test specimens were analyzed using a Q800 dynamic mechanical analyzer TA Instruments (New Castle, DE, USA). The analysis was performed in the range of 30 °C to 120 °C, using a frequency of 1 Hz and a heating rate of 5 °C/min. Here, the Tg was considered as the maximum of the Tan δ peak.

### 2.8. Thermogravimetric Analysis (TGA)

The thermal stability of the biobased crosslinked polymers was determined using a TA Instruments TGA Q500 (New Castle, DE, USA). Samples of 3–5 mg were accurately weighed and placed in the balance chamber of the equipment and analyzed at temperatures ranging from 30 °C to 600 °C at a heating rate of 10 °C/min, in a high-purity nitrogen atmosphere with a flow rate of 50 mL/min.

## 3. Results and Discussion

### 3.1. Synthesis of Curing Agent LCA

**Scheme 1:**
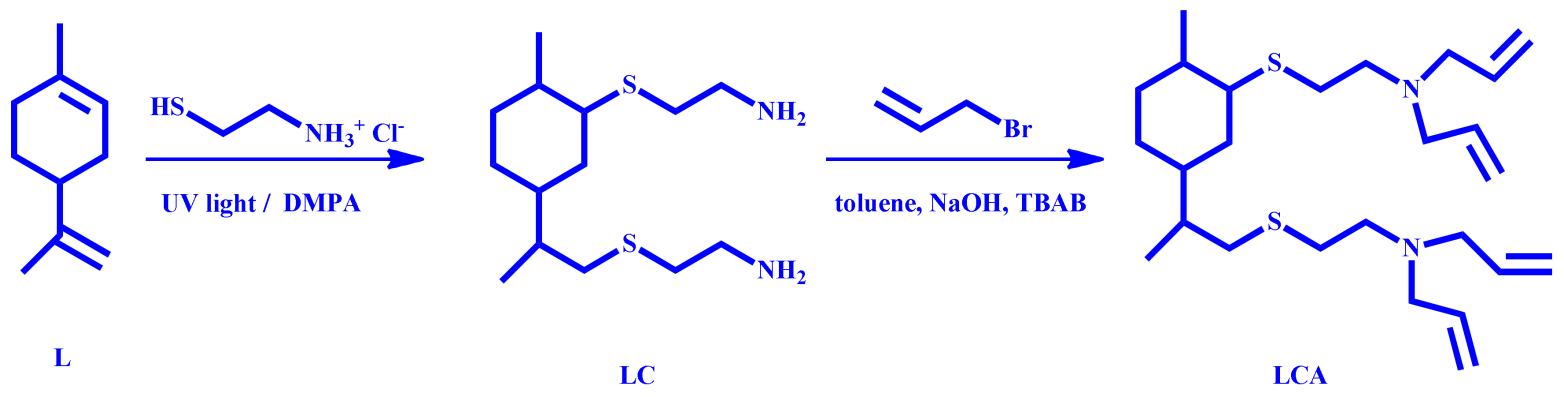
Synthetic methodology to obtain the curing agent LCA.

In this study, a novel curing agent derived from limonene was synthesized with the aim to perform the ETE photopolymerization of the biobased commercial epoxy resin SR GreenPoxy 28 of the Sicomin Company. The curing agent LCA possesses two tertiary amine groups functionalized with allyl groups in its structure. The presence of two double bonds in limonene allows the introduction of primary amine groups, using cysteamine hydrochloride as an aminating agent, by means of a thiol-ene coupling reaction. Once the diamine intermediate (LC) was obtained, it was alkylated using allyl bromide, to achieve the desired curing agent LCA as depicted in Scheme 1.

The difference in reactivity of the endo double bond in the cycle has been compared with the exocyclic double bond of limonene when subjected to thiol-ene coupling reaction. [59]. Moreover, Mattar et.al., reported that the amination of limonene with cysteamine hydrochloride resulted in a mixture of the diaminated and monoaminated limonene [44]. However, regardless of prolonged irradiation times, it was difficult to functionalize the endo double bond with this method, even if an excess of cysteamine hydrochloride was used. Therefore, a new method was essayed where a high intensity of the UV irradiation and a higher concentration of the photoinitiator, were employed. In this way, we obtained the desired intermediate compound in quantitative yield. A mixture of two enantiomers coded as LC was obtained as shown in the chromatogram acquired by GC-MS (Figure S1) and their corresponding mass spectra (Figure S2). LC was reacted further with allyl bromide via a nucleophilic substitution reaction to acquire the curing agent LCA. The reaction mixture, including LC and allyl bromide in a basic medium, was refluxed in toluene under phase transfer catalyst conditions. Following the purification, the compound LCA was achieved as a mixture of enantiomers. Figure S3 presents the chromatogram of the mixture that shows two peaks at a relationship of 48:52 as determined by the percentage area. The mass spectra of both compounds show a molecular ion at 450 um that corresponds with the expected molecular weight. Figure S4 depicts the corresponding mass spectra of both enantiomers.

**Figure 1:**
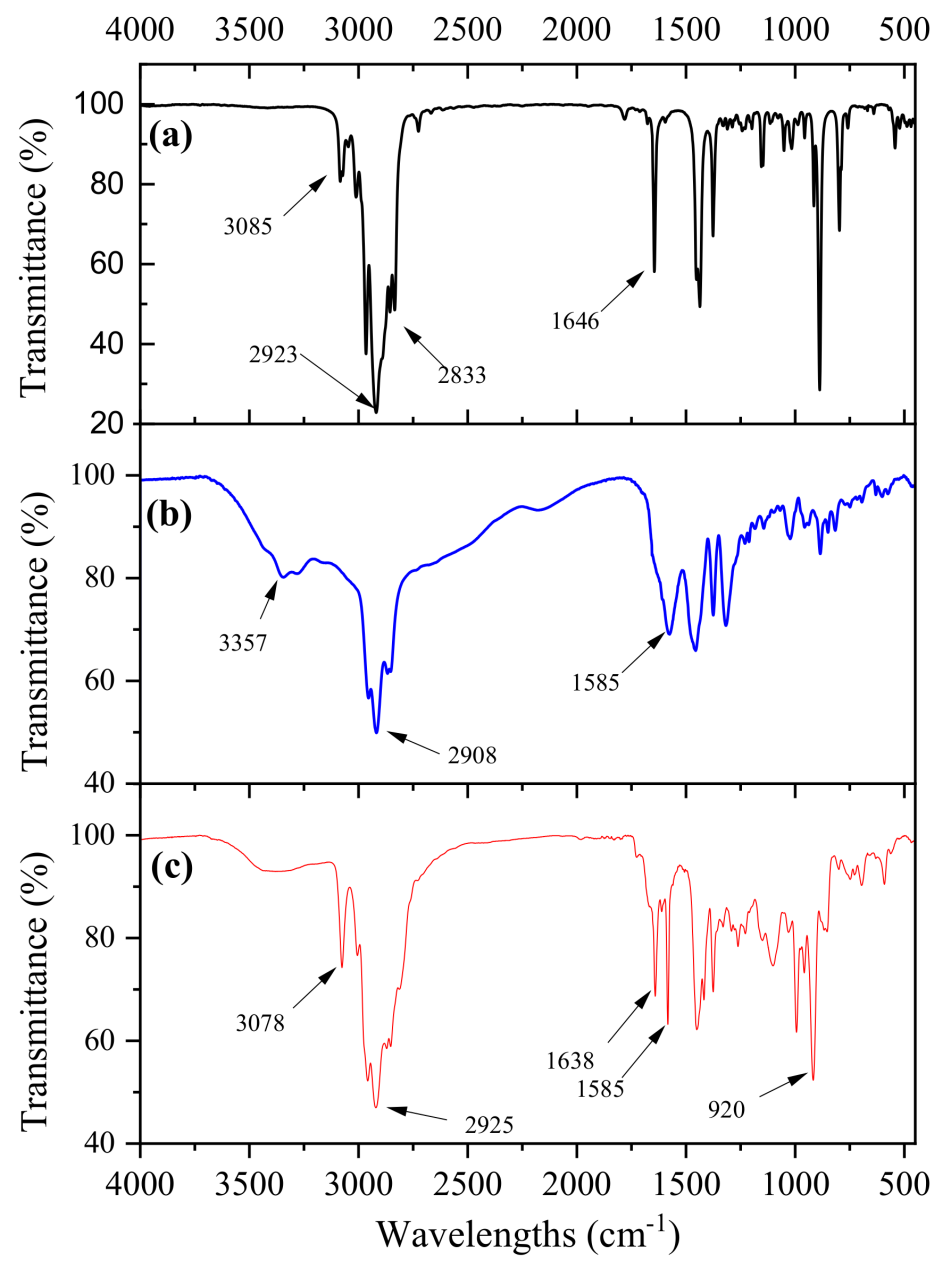
Comparison of the FTIR spectra of: (**a**) pristine limonene, (**b**) intermediate LC compound and (**c**) curing agent LCA. All samples were run as films on a KBr disc.

Figure 1 illustrates a comparison of the FTIR spectra of pristine limonene, the intermediate compound LC and the final compound LCA. 

Following the amination reaction of limonene with cysteamine hydrochloride, the double bond of the limonene at 3085 cm^−1^ and 1646 cm^−1^ (Figure 1a) were not visible in the intermediate compound LC, whereas new bands characteristic of primary amines at 3357 cm^−1^ and of the C-N bond at 1585 cm^−1^ were visible (Figure 1b). This demonstrated the successful functionalization of limonene with primary amine groups. The alkylation of LC with allyl bromide resulted in the obtention of LCA and the bands at 3078 cm^−1^, 1638 cm^−1^ and 920 cm^−1^ confirm the presence of the double bonds in the structure of the desired curing agent, while the band at 1585 cm^−1^ corroborates the presence of C-N bond of the tertiary amine groups (Figure 1c).

**Figure 2:**
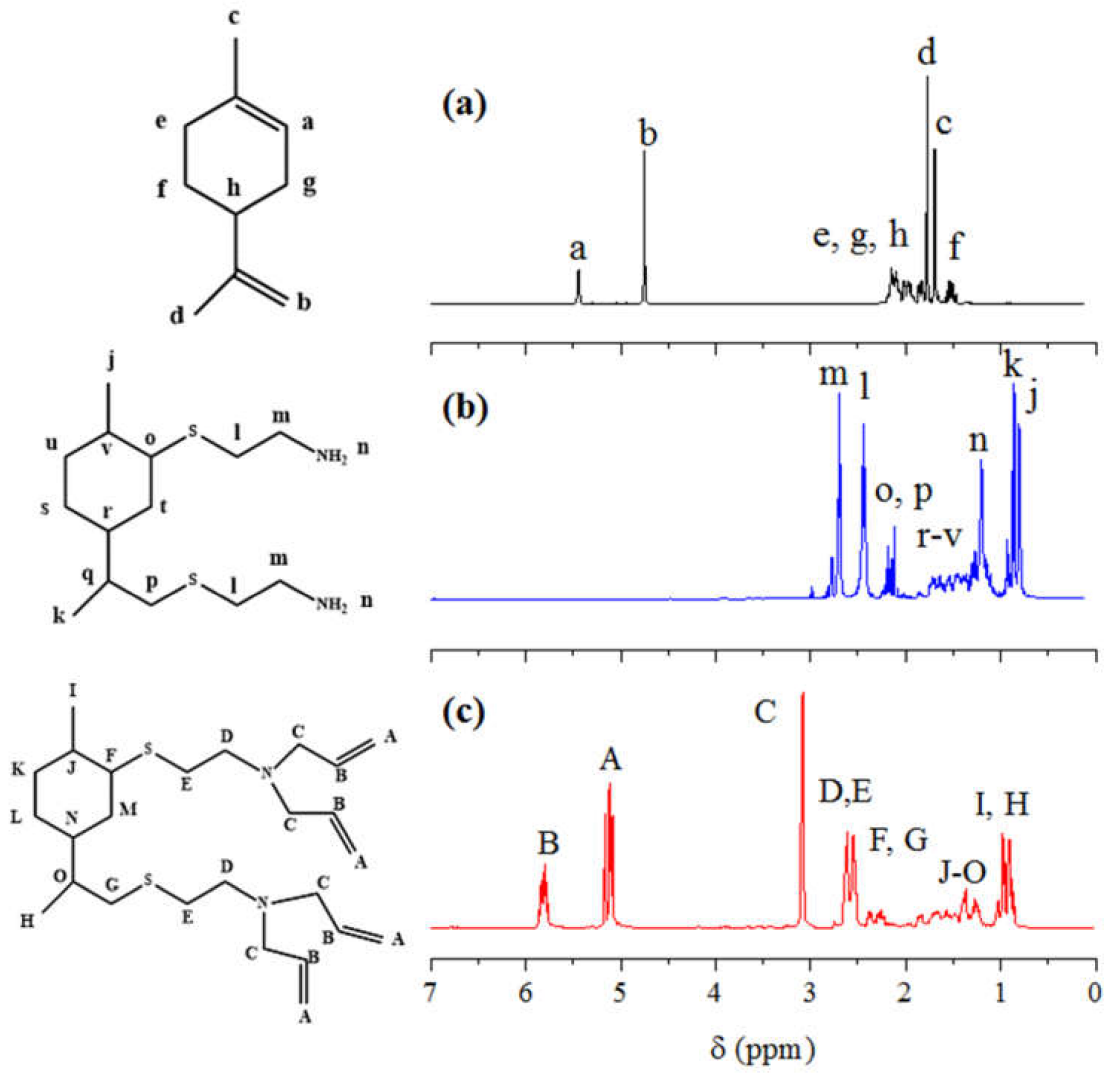
Comparison of the ^1^H NMR spectra of: (**a**) pristine limonene, (**b**) intermediate LC compound and (**c**) curing agent LCA. All samples were run in CDCl_3_ in a 500 MHz spectrometer.

Figure 2 shows the characterization of the compounds by ^1^H NMR spectroscopy. A comparison of the spectra of limonene, LC and LCA is depicted as follows. It can be observed that following the amination reaction of limonene with the cysteamine hydrochloride, the peaks of the internal and external double bonds present in limonene (Figure 2a) at 5.44 ppm and 4.75 ppm, respectively, no longer appear in the intermediate compound LC (Figure 2b) depicting the efficiency of the reaction conditions. The intermediate compound presents two peaks: one at 2.60 ppm and the other at 2.34 ppm which correspond to the protons of the methylene groups adjacent to the tertiary amine group and to the sulfide group, respectively. The spectrum also shows the protons of the cycloaliphatic ring ranging from 1.80 to 1.09 ppm. The protons of the primary amine groups appear centered at 1.20 ppm, overlapped with those of the cycloaliphatic ring. The methyl groups external to the cycloaliphatic ring appear as doublets at 0.77 ppm and 0.71 ppm, due to adjacent methine, in both cases. The ^1^H spectrum of LCA shown in Figure 2c presents the appearance of new peaks at 5.82 ppm and at 5.13 ppm which is characteristic of the double bonds, while a peak at 3.09 ppm corresponds to the allylic protons. The methylenes adjacent to the tertiary amine and to the sulfide group appear as two multiplets centered at 2.63 ppm and 2.53 ppm, respectively. The protons of the cycloaliphatic ring are placed in the range of 1.98–1.02 ppm whereas the protons of the methyl groups appear as doublets located at 0.96 ppm and 0.90 ppm. 

### 3.2. Kinetics of Photopolymerization by RT-FTIR

**Figure 3:**
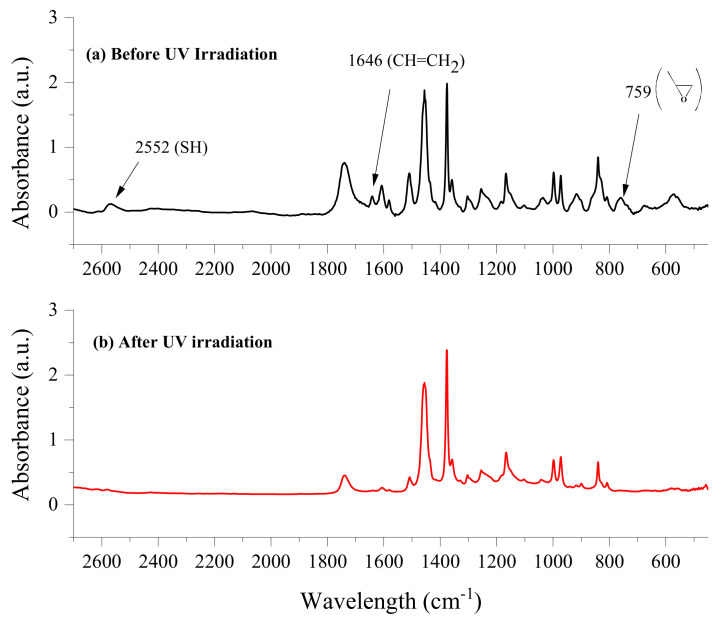
Comparison of the FTIR spectra of the ETE formulation with 40 mol % of TES: (**a**) before and **(b**) after being irradiated with UV light of 40 mW/cm^2^ and 85 °C for 600 s.

The TES was formulated with LCA and PTKMP at a 1:1 ratio—considering that both compounds are tetrafunctionals—and BAPO at 1 mol % in relation to PTKMP. Thereafter, the TES was added to the epoxy resin at 30% and 40 mol %. Previous studies demonstrated that the ETE formulations with those concentrations of TES in the epoxy resin, displayed the highest reactivity [40,47] and the slightly lower modulus in the co-network due to the presence of the flexible polythioethers, which is balanced with the gain in toughness [43]. The samples were irradiated with UV light in the compartment of the FTIR spectrometer and the decrease in the absorbance of the bands corresponding to the epoxy, thiol and double bonds was analyzed. Figure 3 depicts a comparison of the FTIR spectra of the ETE formulation at 40 mol % of TES, before and after 600 s of UV irradiation. It can be observed that the band for the thiol groups at 2552 cm^−1^ as well as the bands corresponding to the double bonds and epoxy groups at 1646 cm^−1^ or 759 cm^−1^, respectively, completely disappeared after the specified irradiation time. 

**Figure 4:**
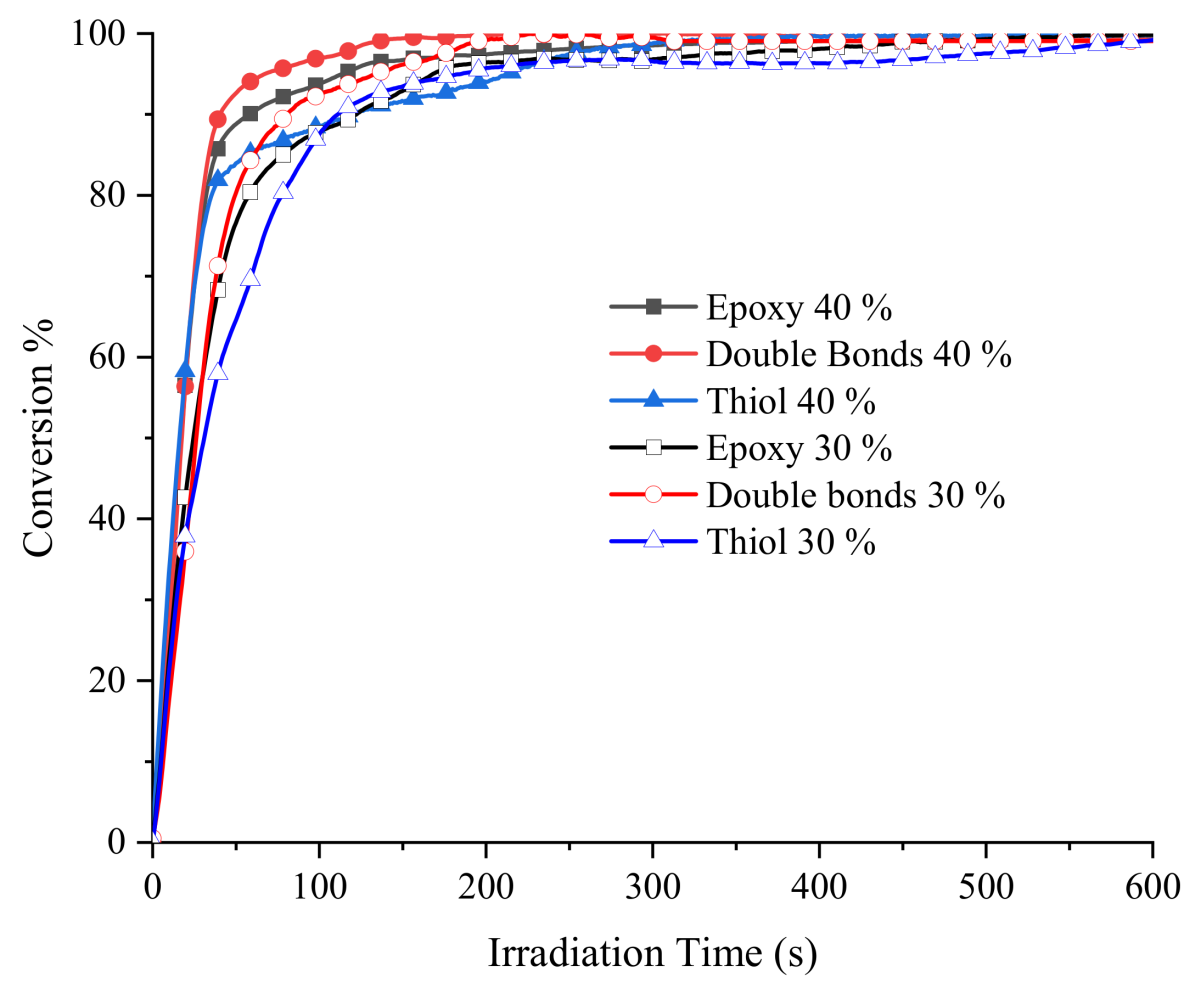
Conversion vs times curves for epoxy groups, thiol groups and double bonds when the TES was added to the epoxy resin at 30 mol % and 40 mol %. The samples were irradiated at 40 mW/cm^2^ and 85 °C using 1 mol % of DMPA.

A more detailed description of the behavior of the functional groups during the photopolymerization can be observed in Figure 4 where the obtained conversion versus time curves is shown for every functional group in the ETE formulations. All functional groups reacted rapidly at both concentrations of the TES, achieving high conversions in the first 60 s of irradiation which demonstrates the high reactivity of this photocurable system. Table 2 summarizes the data obtained from the graph. The functional groups corresponding to the components of the formulation at 40 mol % of TES reacted more rapidly (~ 2.25 s^−1^) than in the case of the formulation with 30 mol % (~1.67 s^−1^), as determined by the slope of the curves. It can be seen that the epoxy groups and double bonds displayed similar curves, achieving 90% and 95% conversion after 60 s of irradiation, whereas 85% of the thiol groups were converted in the same period. After 600 s a quantitative conversion was observed in all cases. 

The conversions achieved by functional groups at 30 mol % of TES, were 85%, 80% and 71% for double bonds, epoxy groups and thiol groups, respectively, in the first min of irradiation, with the quantitative conversion after 600 s. The difference in reactivity is attributed to the higher concentration of the basic initiating species such as the tertiary amine and thiolate groups, in the formulation with 40 mol % of the TES. 

**Scheme 2:**
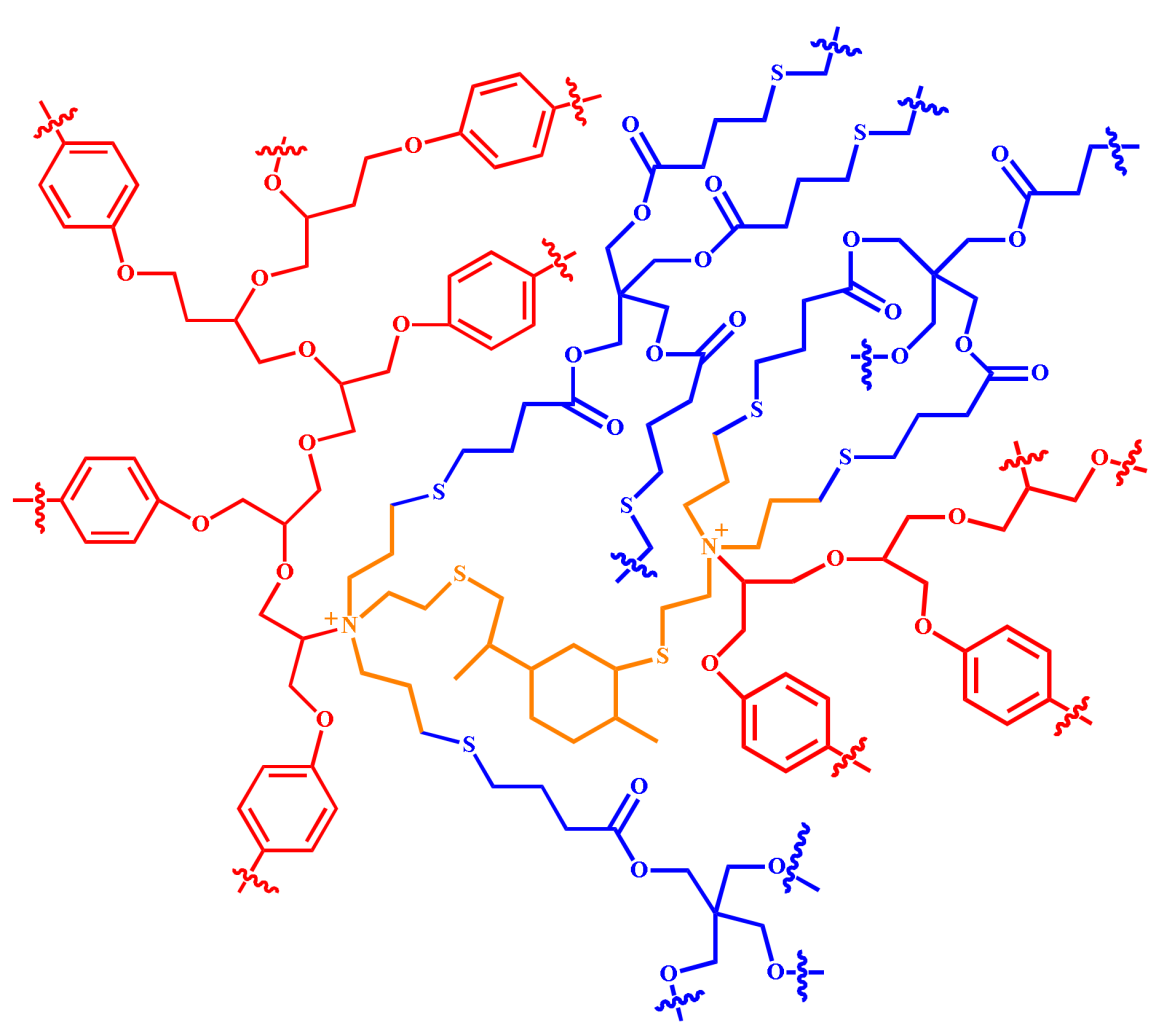
Simplified representation of the polyether-polythioether co-network obtained from the ETE photopolymerization using LCA.

Although theoretically the thiol and double bonds curves should be identical, it can only occur if these groups were involved in the thiol-ene phtopolymerization. However, the proton of the thiol groups can also be extracted by the tertiary amines to produce thiolates which have the potential to attack the epoxy groups to induce their AROP. The proposed mechanism for the ETE photopolymerization has been described in previous works [43,47]. Briefly, the tertiary amines present in LCA can attack the epoxy groups opening the ring, to generate alcoxides which can further attack other epoxy groups to induce the AROP producing polyethers. Simultaneously, the double bonds in LCA can react with the thiol groups of PTKMP to form polythioethers. A secondary reaction may occur when the tertiary amine extracts the hydrogen proton of the thiol groups to form thiolates. These nucleophilic species can also induce the AROP of the epoxy resin to generate polyethers. The produced polythioethers are also basic species—due to the unshared pair of electrons of the sulfur atom—that can also participate in the AROP of the epoxy group. Both the generated polyether and polythioether co-networks are chemically bonded. Scheme 2 depicts a simplified representation of the obtained crosslinked co-network. The red represents the polyether derived from the AROP of the epoxy groups whereas the blue shows the polythioethers originated from the thiol-ene photopolymerization of the double bonds of LCA with the thiol groups of PTKMP. The LCA structure is in the center of the co-network and is colored in orange. The polythioethers are flexible species that inherently impart toughness to the co-network. This property can be modulated by adjusting the concentration of the thiol-ene system in the photocurable formulation. 

### 3.3. Thermal and Thermomechanical Analysis of Obtained Polymers

**Figure 5:**
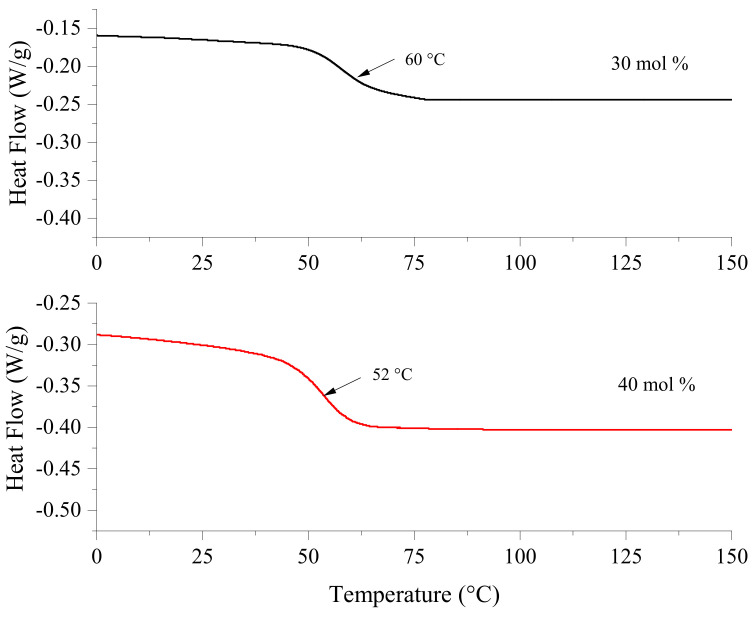
DSC thermograms of the polymers obtained by photopolymerizing formulation with 30 mol % and 40 mol % of TES including the curing agent LCA.

The obtained polymers were analyzed by DSC. Figure 5 depicts the thermograms of the crosslinked co-networks of polyether-polythioether derived from the formulations with 30 mol % and 40 mol % of TES. It can be observed that the former polymer displayed a Tg of 60 °C whereas for the latter the Tg was recorded to be 52 °C. The relatively low Tg in both cases was attributed to the presence of the flexible polythioethers in the co-network, which imparts a certain degree of mobility to the crosslinked network. It can also be hypothesized that the methyl groups in limonene can create free volume in the crosslinked polymer that results in decreased Tg. No other transition was observed in the range of the temperatures analyzed (0–150 °C).

**Figure 6:**
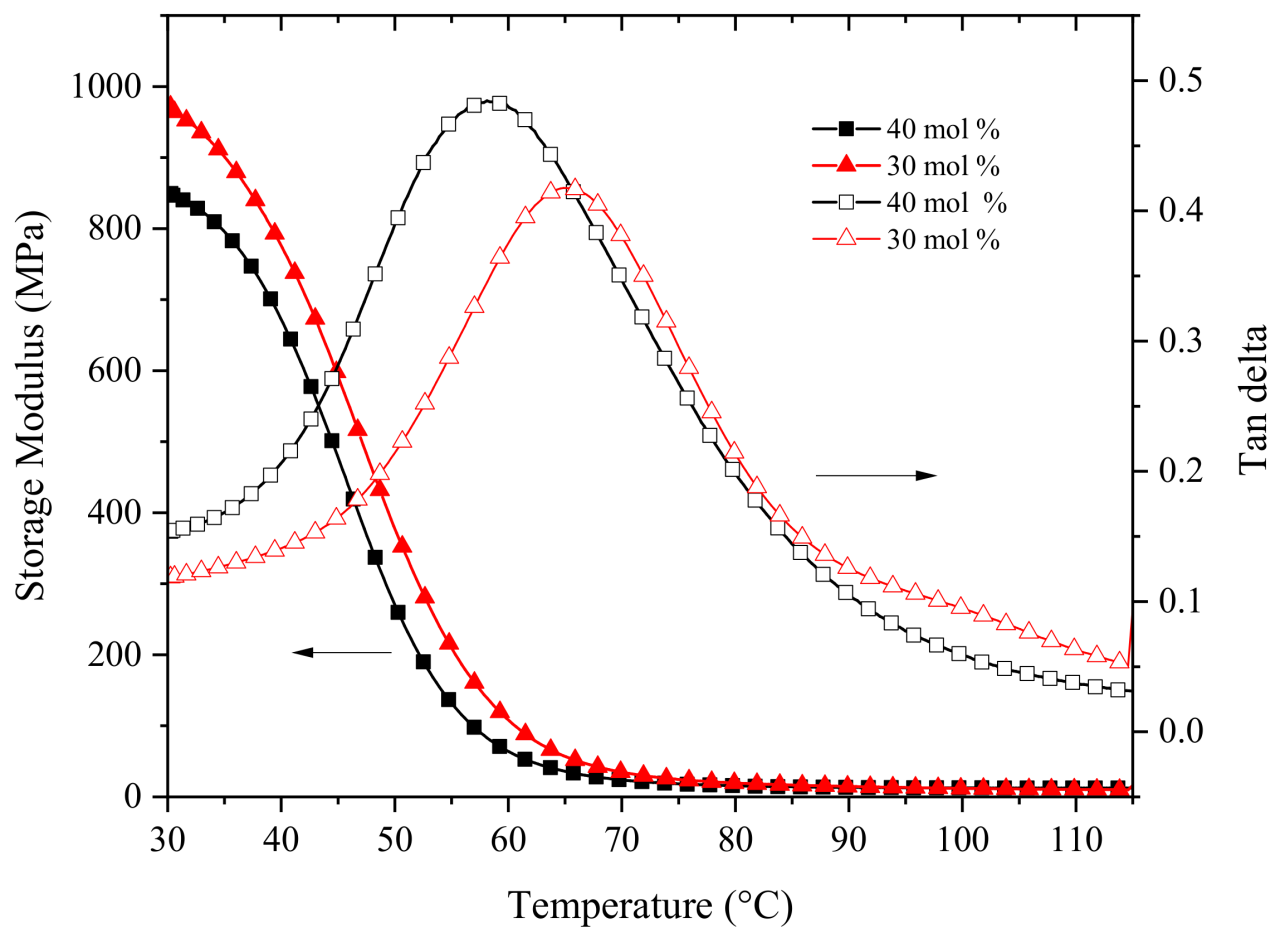
Results of DMA analysis of the polymers obtained by photopolymerizing ETE formulations with 40 mol % and 30 mol % of TES.

The viscoelastic properties of the obtained thermosets polymers were analyzed by DMA and the results are presented in Figure 6. The co-network derived from the ETE formulation with 40% mol of polythioethers displayed a storage modulus of 849 MPa at room temperature and a Tg of 58 °C whereas for the polymer with 30 mol % the modulus was 972 MPa while the Tg was found to be 66 °C. These differences in moduli and Tg can be attributed to the presence of a higher concentration of the flexible polythioethers in the crosslinked co-network with 40 mol % of polythioether relative to that of the co-network with 30 mol %. The obtained values of modulus were slightly lower than those previously reported for a similar ETE formulation using a linear curing agent with two allyl-functionalized tertiary amine groups joined by six methylene units [43]. This can be attributed to the cycloaliphatic backbone of LCA that may hinder the network reticulation to a certain extent leading to a lower modulus than in the case of the linear curing agent [38].

**Figure 7:**
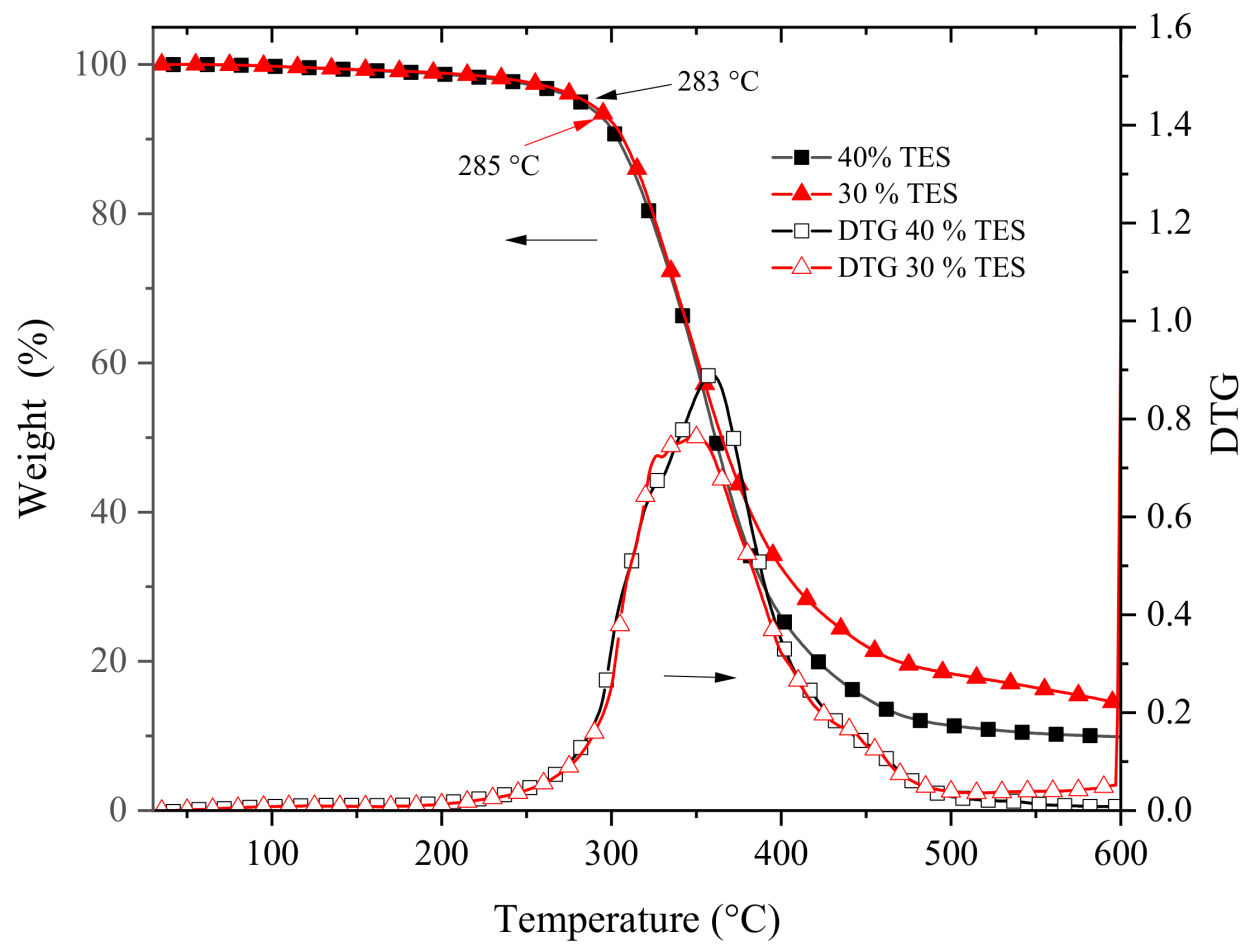
Comparison of the thermograms of the crosslinked polymers derived from the photocurable formulation of the epoxy resin with 40 mol % of TES and 30 mol % of TES.

The thermal stability of the obtained polymers was determined by TGA analysis, and the results are shown in Figure 7. It can be observed that both polymers displayed excellent thermal stability. This may be due to the chemical stability of both polyethers and polythioethers in the co-network. The thermograms are practically identical till 350 °C, which is reasonable considering that the only difference in both polymers is the percentage of polythioethers in the co-network. The onset of degradation i.e., the temperature at which 5% of the weight is lost, was also similar at 283 °C and 285 °C for the polymers with 40 mol % and 30 mol % of polythioethers, respectively. The curves vary in the region above 400 °C where the polymer with 30 mol % of polythioethers produced a carbonaceous residue of 14.27% at 600 °C while the polymer with 40 mol % of polythioethers produced a residue of 9.85% at the same temperature. The DTG curves were also identical till 350 °C, however, above this temperature, both curves displayed a bimodal behavior that was more pronounced for the polymer with 40 mol % of polythioethers. This behavior can be attributed to the differences in thermal stability of polyethers and polythioethers.

## 4. Conclusions

A curing agent derived from limonene was synthesized and used to prepare thermoset crosslinked co-networks by using a commercial biobased epoxy resin. The epoxy/thiol-ene photopolymerization technique was utilized to obtain the biobased polymers in quantitative yield in 20 min. The presence of the allyl-functionalized tertiary amine groups in the curing agent simultaneously promoted the anionic ring-opening polymerization of the epoxy groups as well as a thiol-ene photopolymerization between the allylic groups of the tertiary amine and thiol groups of PTKMP. Furthermore, a polyether-polythioether crosslinked co-network was obtained. The storage moduli for the polymers derived from 30 mol % and 40 mol % were 972 MPa and 849 MPa, respectively. Therefore, the toughness of these materials can be modulated just by varying the ratio of polythioethers in the co-network. These kinds of polymers could have potential applications as coatings.

## Figures and Tables

**Table 1:** Components of the epoxy/thiol-ene photocurable formulations with epoxy resin Greenpoxy 28, LCA, PTKMP and DMPA.

Formulation 30 mol % TES	Equivalents	Moles	Grams
Greenpoxy 28	1	7.3 × 10^−4^	0.25
LCA	0.3	2.2 × 10^−4^	0.10
PTKMP	0.3	2.2 ×10^−4^	0.11
DMPA	0.003	2.2 × 10^−6^	5.6 ×10^−3^
Formulation 40 mol % TES	Equivalents	Moles	Grams
Greenpoxy 28	1	7.3 × 10^−4^	0.25
LCA	0.4	2.9 × 10^−4^	0.13
PTKMP	0.4	2.9 ×10^−4^	0.15
DMPA	0.004	2.9 × 10^−6^	7.4 × 10^−3^

The formulations were photopolymerized in a UV chamber using a UV light intensity of 40 mW/cm^2^. and 85 °C during 20 min.

**Table 2:** Summary of conversions and photopolymerization rates of double bonds of LCA, epoxy groups of SR GreenPoxy 28 and thiol groups of PTKMP during the ETE photopolymerization.

Formulation	Double Bonds (1646 cm^−1^)			Epoxy Groups (867 cm^−1^)			Thiol Groups (2552 cm^−1^)		
	R_p_/M_0_ * (s^−1^)	Conversion (%)		R_p_/M_0_ * (s^−1^)	Conversion		R_p_/M_0_ * (s^−1^)	Conversion (%)	
		60 s	600 s		60 s	600 s		60 s	600 s
40 mol % TES	2.25	95	99	2.17	90	99	2.08	85	99
30 mol % TES	1.67	85	99	1.52	81	99	1.50	70	99

* Determined at the slope of the curve.

## Data Availability

Not applicable.

## Supplementary Materials

The following supporting information can be downloaded at: https://www.mdpi.com/article/10.3390/polym14112192/s1, Figure S1: Chromatogram of compound LC; Figure S2: Mass Spectra of enantiomers LC; Figure S3: Chromatogram of compound LCA; Figure S4: Mass Spectra of enantiomers LCA.
