# Supplementary material for: Synthesis of a Curing Agent Derived from Limonene and the Study of Its Performance to Polymerize a Biobased Epoxy Resin Using the Epoxy/Thiol-Ene Photopolymerization Technique
Source: Polymers (Basel). 2022 May 28;14(11):2192. doi: 10.3390/polym14112192 (PMC9182678; doi:10.3390/polym14112192)
Supplement: Supplementary file 1 [file polymers-14-02192-s001.zip › polymers-1739681-supplementary.pdf]

Supplementary material for paper

Synthesis of a Curing Agent derived from Limonene to polymerize a biobased epoxy resin using the Epoxy/Thiol-Ene Photopolymerization technique

Ricardo Acosta Ortiz*, Rebeca Sadai Sánchez Huerta, Antonio Serguei Ledezma Pérez, Aida E. García Valdez

Centro de Investigación en Química Aplicada, Blvd Enrique Reyna #140, Saltillo, Coahuila, México Z.C. 25294 email: ricardo.acosta@ciqa.edu.mx

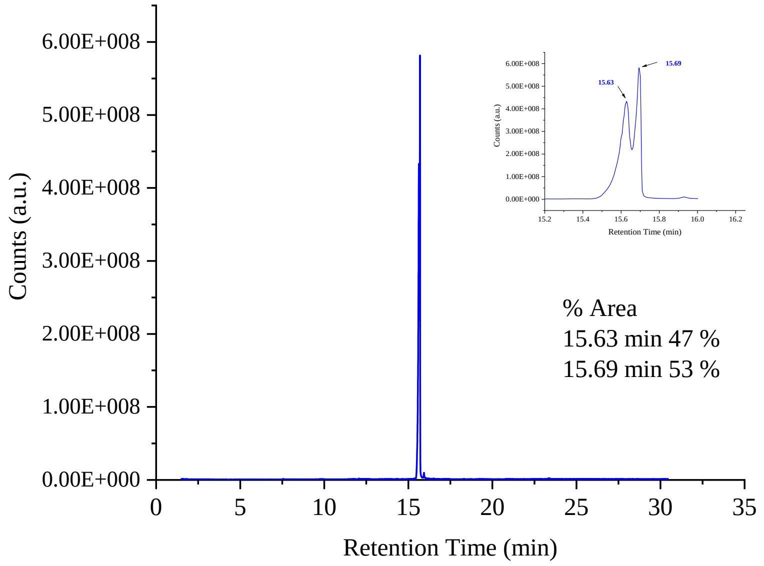

Figure S1. Chromatogram of compound LC run in a Thermofisher GC-MS Chromatograph model 1310 equipped with a column Thermo Tg-SQC 30m * 0.25mm * 0.25um. The temperature of the injector was 250°C with a nitrogen flow of 1.5 mL/min. The running conditions were 100°C -3.0 min with a ramp of 15°C/min up to 300°C. The overall time of the run was 30 min. The mass detector was a single quadrupole mass spectrometer ISQ-LT, with a MS transfer line temperature of 250°C and an ion source temperature of 200° C

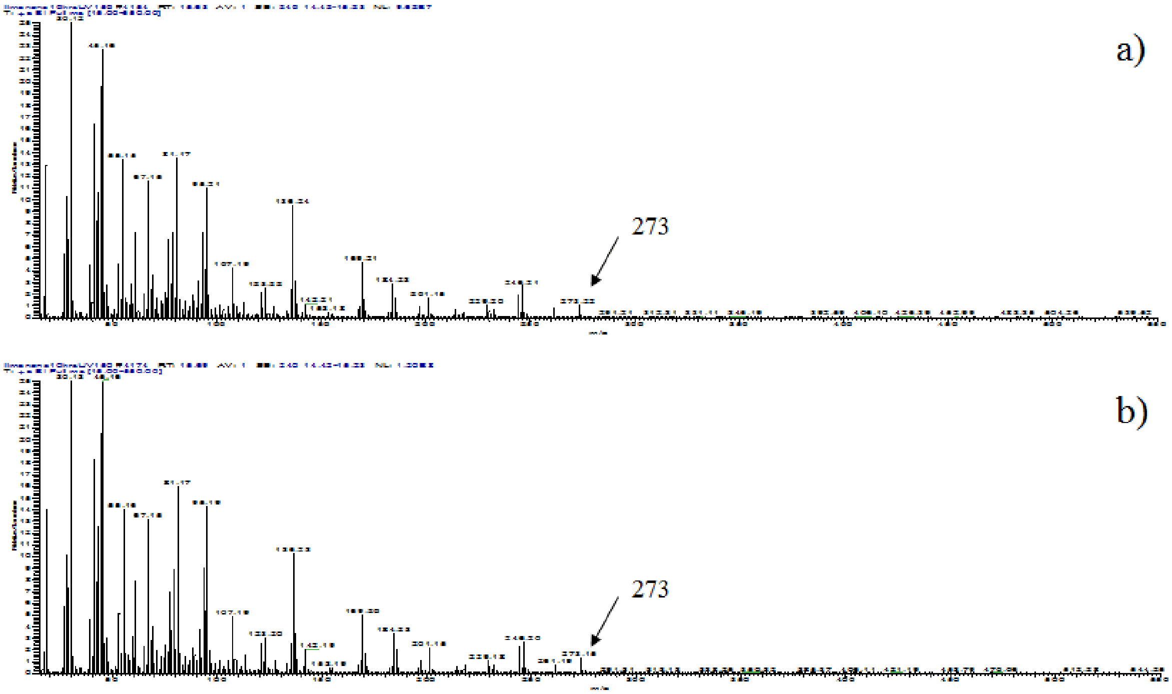

Figure S2. Mass spectrum of enantiomers LC: a) compound at retention time of 15.63 min, b) compound at retention time of 15.69, peak at 273 correspond to the loss of one amino group ($-NH_2$) of the compound LC

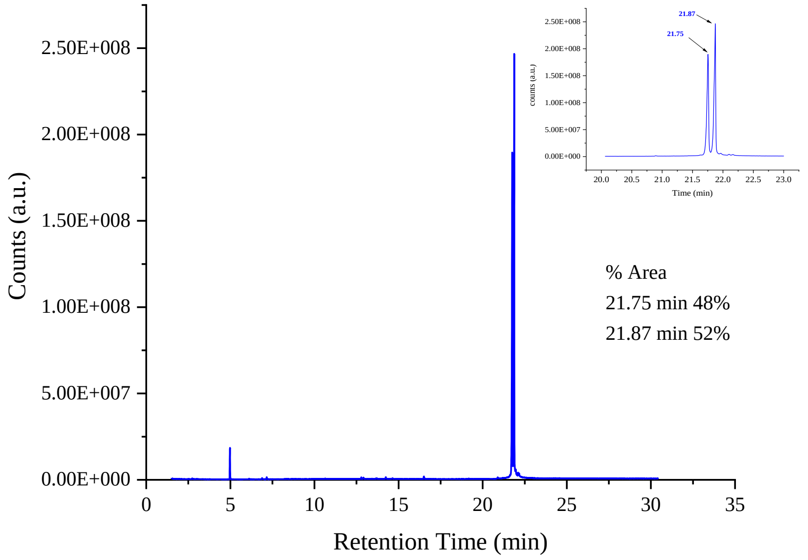

Figure S3. Chromatogram of compound LCA run in a Thermofisher GC-MS Chromatograph model 1310 equipped with a column Thermo Tg-SQC 30m * 0.25mm * 0.25um. The temperature of the injector was 250°C with a nitrogen flow of 1.5 mL/min. The running conditions were 100°C -3.0 min with a ramp of 15°C/min up to 300°C. The overall time of the run was 30 min. The mass detector was a single quadrupole mass spectrometer ISQ-LT, with a MS transfer line temperature of 250°C and an ion source temperature of 200°C

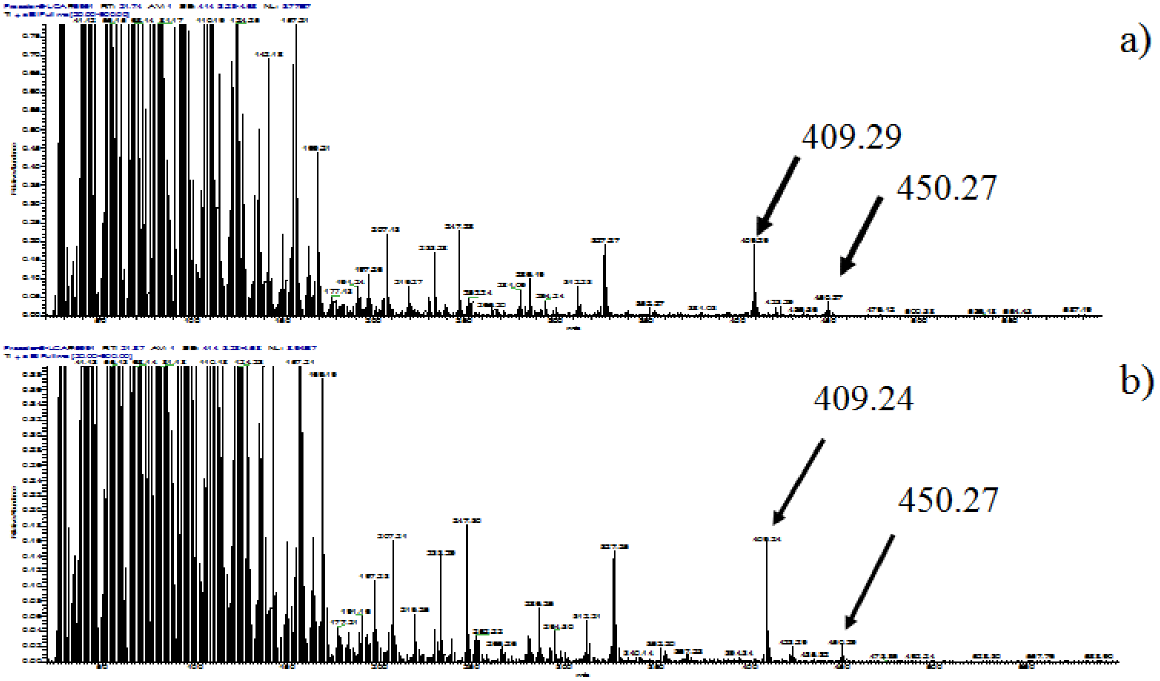

Figure S4. Mass spectra of enantiomers LCA: a) compound at retention time of 21.75 min, b) compound at retention time of 21.87, peak at 450.27 correspond to molecular ion peak, and peak at 409.24 correspond to the loss of the allylic group ($-CH_2-CH=CH_2$)
